# Metropolitan Hospital Sunday Fund

**Published:** 1904-08-13

**Authors:** 


### HOSPITAL ADMINISTRATION.

### CONSTRUCTION AND ECONOMICS.

### METROPOLITAN HOSPITAL SUNDAY FUND.

### COUNCIL MEETING.

The Lord Mayor, Sir James Thomson Ritchie, Bart., presided over a meeting of the Council of the Metropolitan Hospital Sunday Fund, at the Mansion House, on Tuesday, August 9th, for the purpose of receiving the report of the Committee of Distribution and recommending that the payment of the awards should be made as soon as possible.

Letters of regret had been received from the Bishops of London and Stepney, the Chief Rabbi, Mr. Cosmo Bonsor, and others, who were unavoidably absent.

The Chairman moved the adoption of the report, alluding to the regret felt by the council on account of the death of Mr. Herman Hoskier, who had been an active member of the committee since 1888. In their report last year, he said, the committee called the attention of the council to the constantly increasirg expenditure per occupied bed at some of the hospitals. This year the committee were glad to note that the conference which had taken place with the committees of these hospitals had in several cases resulted in a considerable reduction in expenditure being effected. Awards were now recommended to 215 institutions, as shown in the appendix attached to the report.

#### The Year's Work.

The amount of collections, including interest from the Court of Chancery to the present time, was £59,360. The distribution of £56,371 8s. 4d. to 157 hospitals and 58 dispensaries was recommended. The Lord Mayor drew special attention to the committee's desire to be assured that in all institutions to which grants were made adequate precautions were taken for the safety of patients in case of fire. This year 218 institutions had made applications for grants from the Fund—*i.e.* seven more than in 1903—and just double the number that applied in 1874. The Committee did not recommend an award to the City Orthopædic Hospital, being of opinion that the governors should accept the proposals of King Edward's Hospital Fund, and amalgamate with the two other orthopædic hospitals. The committee, the Lord Mayor remarked, could not quite understand why this hospital should not amalgamate. If the authorities would agree to do so, the hospital would be placed in comfortable circumstances, and a considerable grant would be received from King Edward's Hospital Fund. The matter had been thoroughly gone into, but the authorities apparently preferred to remain in Hatton Garden, especially as many of the patients were children, a course which he could not think so desirable as amalgamation, with a country branch as one feature of development. He could not conclude without a warm tribute of thanks to Mr. George Herring, who had again contributed a sum equal to one-fourth of the total collected in public places of worship.

Sir Sydney H. Waterlow, in seconding, said that although the collection was not so large as last year—the "King's year"—yet he thought on the whole there was ample cause for gratitude. The amount, moreover, would be slightly augmented, since the financial year did not close until October 31st, by which time several outstanding collections would be received.

#### £1,306,000 Collected.

It should never be forgotten, he remarked, that the total collected in the 32 years of the Fund's existence was £1,306,000. Members of the council, and the public generally, appreciated the progress made from year to year, progress which constituted the best possible guarantee that the public were satisfied with the method of distribution. The number of institutions applying for grants had exactly doubled since the Fund was started. The reduction in this year's total was, he thought, due to smaller sums being contributed by some of the richer churches, the reduction not being in the same proportion in the collections from churches in poorer neighbourhoods. Very great care had been taken by the committee in making inquiries into the general condition and management of every hospital making application, both by personal visits, and by conferring with deputations, 12 of which had been received, in reference to reduced bases of award. St. Thomas's Hospital had made no application, because the authorities considered they did not need a grant, but the committee were of opinion that, in the future, some outside assistance would be required by that hospital. With reference to the City Orthopædic Hospital, copies of an extract from the report of King Edward's Hospital Fund were in the hands of the meeting, and he would only remark that the two funds were in perfect agreement and had always worked in entire harmony. The Distribution Committee, he said, were of opinion that one first-rate orthopædic hospital, with its country branch, would tend more to the general benefit of this class of patients than a number of institutions in London in rivalry with one another; they regretted that the City Orthopædic Hospital had not at present seen its way to join in any scheme for amalgamation.

Sir Henry Burdett, K.C.B., speaking on the report, congratulated the Committee of Distribution on the general results of the collection in a year, which, in a business sense, was probably the worst the City of London had experienced within living memory. That, he thought, was not too strong a statement. As representing the lay element, he took the opportunity of expressing their sense of gratitude to the clergy of all denominations, to whose exertions this success in a bad year was eminently due. Proceeding to comment on the paragraph in the report which alluded to the tendency to ever-increasing expenditure per occupied bed in some of the hospitals, Sir Henry said there could be no doubt that each hospital committee should nowadays fix a maximum unit of expenditure per bed.

#### A Plea for Medical Control.

The fact was that the tendency had been continually upwards, the increase being, he believed, partly due to the want of appreciation of the importance of having absolute control inside over every department of a great hospital. In going about the country inspecting hospitals, he was immensely impressed by the evidence of efficiency and control in the provincial, and especially in the Scottish hospitals, where there was a medical superintendent in charge of every department. The greater the number of nurses, and the larger the medical schools, the greater the tendency to waste in such matters as surgical and medical dressings, and other expensive items, and he wished to emphasise the fact that in those London hospitals where there was a medical superintendent charged with controlling a medical department of expenditure, a much smaller expenditure was shown than in those where there was no such superintendent. It might be contended that the expenditure under the head of salary—running perhaps to £500 or £600 a year or more if keep were included—was great, but he would show that large economy was effected in the result. For instance, taking two large hospitals, equally important, the one without a medical superintendent spent £70 a week more than the one with departmental control, while a third, also without a medical superintendent, spent £150 more per week. The annual increased cost, therefore, was £3,640 in the first case and £7,800 in the second. He did not think it necessary to detain the Council long in enforcing this point, because the figures were too eloquent to be explained away; he would content himself with drawing their attention to the facts.

With reference to the City Orthopædic Hospital, he was not aware how many members of the Council were familiar with it, but if any one else had any doubt as to the wisdom and necessity of the decision arrived at by the Distribution Committee, he could only say that the best answer to such criticism was to invite the subscribers and the public generally to pay a visit to that hospital. He was quite sure they would agree with every expert opinion—and there had been a good many visits paid by experts since the suggestion was made to amalgamate—that as to its equipment and entire buildings, it was not in a condition which the medical profession would consider adequate to the treatment of acute medical and surgical cases. Let anybody, whether familiar with hospitals or not, go and lie, as he had done, on one of the beds, and think what it must mean to a patient to have to lie there for eight or ten weeks, with nothing to look at but a blank wall. In the belief that sometimes frankness was the essence of justice, he would say plainly that not public but private considerations prevented that hospital from coming into the scheme. Everyone who believed in the adequate and up-to-date equipment and efficiency of the hospitals of London, in order that surgical and medical care might be available for every poor person who required it, must combine to force the City Orthopædic Hospital to alter its decision, and to come in and join hands with the Royal and the National Orthopædic Hospitals, and to make for London what was needed, *i.e.* a great orthopædic hospital.

The report was adopted unanimously.

Sir Edward Durning Lawrence moved that the cordial thanks of the council be given to Mr. George Herring for his generous and continued interest in the Fund. While thanking Mr. Herring most warmly for his donation of over £11,000 the speaker said that it was not wise that great charities should depend on single individuals. The pence of the poor in the small churches were wanted, as well as the princely gifts of the rich man.

Votes of thanks were also passed to the members of the Committee of Distribution, special mention being made of the labours of Sir Sydney Waterlow, and to the Press. The meeting concluded with a vote of thanks to the Lord Mayor for presiding, and for the time and influence he had devoted as president and treasurer, to the well-being of the Fund.

### DISTRIBUTION COMMITTEE'S REPORT AND AWARDS, 1904.

The Committee of Distribution in submitting their report desire to record with great regret the death of Mr. Herman Hoskier, who had been an active member of this committee since 1888.

The committee in their report last year called the attention of the council to the constantly increasing expenditure per occupied bed at some of the hospitals. The committee are glad to note that the conference which has taken place with the committees of these hospitals, has, in several cases, resulted in a considerable reduction having been effected.

The committee recommend awards this year to the 215 institutions shown in the appendix attached hereto. The amount of collections, including interest from the Court of Chancery, to the present time is £59,360. The distribution of £56,371 8s. 4d. to 157 hospitals and 58 dispensaries is now recommended.

The committee desire to be assured that in all institutions to which grants are made adequate precautions are taken for the safety of patients in case of fire.

This year 218 institutions have made application for grants from the Fund, being seven more than in 1903, and just double the number that applied in 1874.

The committee do not recommend an award to the City Orthopædic Hospital, being of opinion that the Governors of this hospital should accept the proposal of King Edward's Fund, and amalgamate with the two other orthopædic hospitals.

Two hospitals which applied for the first time, were considered ineligible.

The committee having reduced the bases of award in 25 cases, this fact was intimated to each hospital interested in accordance with Rule V. Twelve deputations attended the committe and, after hearing their cases, the final awards were determined.

Mr. George Herring has again most liberally offered to give one-fourth to the amount collected in places of worship.

Five per cent. of the total sum collected is set apart to purchase surgical appliances (in obedience to Law XII.) in monthly proportions during the ensuing year.

In compliance with an order of the council, and for the special use of its members, tables of statistics have been prepared as usual, showing an analysis of the number of beds in hospitals, the cost of patients both at hospitals and dispensaries, the proportionate expense of management, as well as other valuable information, and copies are sent to every member of the council.

### PARTICULARS OF AWARDS.

Particulars of the awards recommended by the Committee of distribution for the year 1904.

### 31 GENERAL HOSPITALS.

Charing Cross Hospital, £987 1s. 8d.; French Hospital, £325 16s. 8d.; German Hospital, £565 8s. 4d.; Great Northern Central Hospital, £1,029 1s. 2d.; Guy's Hospital, £2,041 5s.; Hampstead General Hospital, £204 2s. 6d.; Italian Hospital, £182 1s. 8d.; King's College Hospital, £1,458 11s. 8d.; London Hospital, £4,992 18s. 4d.; London Homœopathic Hospital, £533 15s. 10d.; Phillips' Memorial Homœopathic Hospital, £23 19s. 2d.; London Temperance Hospital, £791 11s. 8d.; Metropolitan Hospital, £902 15s.; Mildmay Hospital, £282 14s. 2d.; Miller Hospital and Royal Kent Dispensary, £253 3s. 8d.; North-West London Hospital, £505 0s. 10d.; Poplar Hospital, £661 5s.; Queen's Jubilee Hospital, £153 6s. 8d.; Royal Free Hospital, £1,341 13s. 4d.; St. George's Hospital, £1,629 3s 4d.; S.S. John and Elizabeth Hospital, £327 15s.; St. Mary's Hospital, £2,395 16s. 8d.; Seamen's Hospital Society, £1,386 14s. 2d.; The Middlesex Hospital and Convalescent Home, £2,501 5s.; Tottenham Hospital, £445 12s. 6d.; University College Hospital, £1,760 9s. 2d.; Walthamstow, etc., Hospital, £98 10s. 9d.; West Ham Hospital, £526 12s. 6d.; West London Hospital, £1,015 16s. 8d.; Westminster Hospital, £1,533 6s. 8d.; St. John's Hospital, Lewisham, £191 13s. 4d.

### 34 SPECIAL HOSPITALS.

#### 5 Chest Hospitals.

City of London Hospital for Diseases of the Chest, Victoria Park, £929 11s. 8d.; Hospital for Consumption, Brompton, £1,820 16s. 8d.; Mount Vernon Consumption Hospital, Hampstead, £766 13s. 4d.; Royal Hospital for Diseases of the Chest, City Road, £431 5s.; Royal National Hospital for Consumption (Ventnor), £335 8s. 4d.

#### 17 Children's Hospitals.

Alexandra Hospital for Hip Disease, W.C., £297 1s. 8d.: Banstead Surgical Home, £23 19s. 2d.; Barnet Home Hospital, £43 2s. 6d.; Belgrave Hospital for Children, S.W., £72 18s. 8d.; Cheyne Hospital for Incurable Children, S.W., £138 19s. 2d.; East London Hospital for Children, Shadwell, E., £805; Evelina Hospital for Sick Children, Southwark, S.E., £95 16s. 8d.; Home for Incurable Children,

Maida Vale, W., £71 17s. 6d.; Home for Sick Children, Sydenham, S.E., £129 7s. 6d.; Hospital for Sick Children, Great Ormond Street, W.C., £1,102 1s. 8d.; Kensington, for Children, and Dispensary, £81 9s. 2d.; North-Eastern Hospital for Children, Hackney Road, N.E., £431 5s.; Paddington Green Hospital for Children, W., £306 13s. 4d.; St. Mary's Hospital, Plaistow, E., £258 15s.; St. Monica's Hospital, Brondesbury, N.W., £67 1s. 8d.; Victoria Hospital for Children, King's Road, Chelsea, S.W., £527 1s. 8d.; Victoria Home, Margate, £40 5s.; Hospital for Hip Disease, Sevenoaks, £43 2s. 6d.

### 6 Lying-in Hospitals.

British Lying-in Hospital, Endell Street, W.C., £43 2s. 6d.; City of London Lying-in Hospital, City Road, E.C., £100; Clapham Maternity Hospital, £19 3s. 4d.; East End Mothers' Home, £67 1s. 8d.; General Lying-in Hospital, Lambeth, S.E., £59 8s. 4d.; Queen Charlotte's Lying-in Hospital, Marylebone Road, W., £450 8s. 4d.

### 6 Hospitals for Women.

Chelsea Hospital for Women, S.W., £273 2s. 6d.; Hospital for Women, Soho Square, W., £327 15s.; Grosvenor Hospital for Women and Children, Vincent Square, S.W., £143 15s.; New Hospital for Women, Euston Road, W.C., £287 10s.; Royal Waterloo Hospital for Children and Women, Lambeth, S.E., £191 13s. 4d.; Samaritan Free Hospital, Marylebone Road, W., £335 8s. 4d.

### 27 OTHER SPECIAL HOSPITALS.

Cancer Hospital, Brompton, S.W., £445 12s. 6d.; Middlesex Hospital, Cancer Wing, £268 6s. 8d.; London Fever Hospital, Islington, N., £598 19s. 2d.; Gordon Hospital for Fistula, Vauxhall Bridge Road, S.W., £17 5s.; St. Mark's Hospital for Fistula, City Road, E.C., £191 13s. 4d.; National Hospital for the Diseases of the Heart, Soho Square, W., £131 5s. 10d.; Female Lock Hospital, Harrow Road, W., £206 0s. 10d.; Hospital for Epilepsy, Paralysis, and other Diseases of the Nervous System, Regent's Park, N.W., £53 13s. 4d.; National Hospital for the Paralysed and Epileptic, W.C., £1,159 11s. 8d.; West End Hospital for Diseases of the Nervous System, £285 15s. 0d.; Central London Ophthalmic Hospital, Gray's Inn Road, W.C., £164 16s. 8d.; Royal Eye Hospital, St. George's Circus, S.E., £292 5s. 10d.; Royal London Ophthalmic Hospital, City Road, E.C., £1,006 5s.; Royal Westminster Ophthalmic Hospital, Charing Cross, W.C., £177 5s. 10d.; Western Ophthalmic Hospital, Marylebone Road, W., £71 17s. 6d.; City Orthopædic Hospital, Hatton Garden, E.C., nil; National Orthopædic Hospital, Great Portland Street, W., £153 6s. 8d.; Royal Orthopædic Hospital, Oxford Street, W., £110 4s. 2d.; Royal Sea Bathing Hospital, Margate, £287 10s.; St. John's Hospital for Diseases of the Skin, £47 18s. 4d.; St. Peter's Hospital for Stone, Covent Garden, £95 16s. 8d.; Central London Throat and Ear Hospital, Gray's Inn Road, W.C., £57 10s.; Hospital for Diseases of the Throat, Golden Square, W., £62 5s. 10d.; London Throat Hospital, Great Portland Street, W., £34 10s.; Royal Ear Hospital, Frith Street, W., £22 0s. 10d.; Royal Dental Hospital of London, W.C., £213 14s. 2d.; National Dental Hospital, 149 Great Portland Street, W., £30 13s. 4d.

### 31 CONVALESCENT HOSPITALS.

Metropolitan Convalescent Institution, Walton, £619 15s. 4d.; Metropolitan Convalescent Institution, Bexhill, £338 14s.; All Saints' Convalescent Hospital, Eastbourne, £383 6s. 8d.; Ascot Priory Convalescent Home, £71 17s. 6d.; Brentwood, for Children, £11 10s.; Charing Cross Hospital Convalescent Home, Limpsfield, £91 0s. 10d.; Chelsea Hospital for Women Convalescent Home, St. Leonards, £38 6s. 8d.; Deptford Medical Mission Convalescent Home, Bexhill, £28 15s.; Mrs. Gladstone Convalescent Home, Mitcham, £86 5s.; Friendly Societies' Convalescent Home, Dover, £76 13s. 4d.; Hahnemann Convalescent Home, Bournemouth, £43 2s. 6d.; Hanwell Convalescent Home, £19 3s. 4d.; Hendon, Ossuston Home, £52 14s. 2d.; Herbert Convalescent Home, Bournemouth, £28 15s.; Herne Bay Baldwin-Brown Convalescent Home, £28 15s.; Homœopathic Hospital Convalescent Home, Eastbourne, £19 3s. 4d.; King's College Hospital Convalescent Home, Hemel Hempstead, £63 5d.; Mrs. Kitto's Convalescent Home, Reigate, £57 10s.; Mary Wardell Convalescent Home for Scarlet Fever, £86 5s.; Morley House Convalescent Home for Working Men, £162 18s. 4d.; Alfred Beavan Memorial, Sandgate, £134 3s. 4d.; Police Seaside Home, Brighton, £52 14s. 2d.; St. Andrew's Convalescent Home, Clewer, £115; St. Andrew's Convalescent Home, Folkestone, £172 10s.; St. John's Home for Convalescent and Crippled Children, Brighton, £28 15s.; St. Joseph's Convalescent Home, Bournemouth, £57 10s.; St. Leonards-on-Sea Convalescent Home for Poor Children, £81 9s. 2d.; St. Mary's Convalescent Home, Shortlands, £20 2s. 6d.; St. Michael's Convalescent Home, Westgate-on-Sea, £33 10s. 10d.; Seaside Convalescent Hospital, Seaford, £95 16s. 8d.; Richmond House, Worthing, £19 3s. 4d.

### 22 COTTAGE HOSPITALS.

Acton Cottage Hospital, £57 10s.; Beckenham Cottage Hospital, £88 3s. 4d.; Blackheath and Charlton Cottage Hospital, £85 15s. 10d.; Bromley, Kent, Cottage Hospital, £115; Bushey Heath Cottage Hospital, £52 14s. 2d.; Canning Town Cottage Hospital, £59 8s. 4d.; Chislehurst, Sidcup, and Cray Valley Cottage Hospital, £52 5s.; Coldash Cottage Hospital, £17 5s.; Eltham Cottage Hospital, £47 18s. 4d.; Enfield Cottage Hospital, £69 19s. 2d.; Epsom and Ewell Cottage Hospital, £74 15s.; Hounslow Cottage Hospital, £29 10s. 8d.; Kingston, Victoria Hospital, £53 1s. 8d.; Livingstone Cottage Hospital, Dartford, £90 1s. 8d.; Mildmay Cottage Hospital, £19 3s. 4d.; Reigate and Redhill Cottage Hospital, £85 8s. 4d.; Sidcup Cottage Hospital, £28 15s.; Tilbury (Passmore Edwards) Cottage Hospital, £38 6s. 8d.; Willesden Cottage Hospital, £125 10s. 10d.; Wimbledon Cottage Hospital, £48 17s. 6d.; Wimbledon South Cottage Hospital, £30 6s. 8d.; Woolwich and Plumstead Cottage Hospital, £47 18s. 4d.

### 12 INSTITUTIONS.

Bolingbroke Hospital, £239 11s. 8d.; Hospital for Invalid Gentlewomen, Harley Street, £138 19s. 2d.; St. Saviour's Hospital and Nursing Home, £115; National Sanatorium for Consumption, Bournemouth, £95 16s. 8d.; Invalid Asylum, Stoke Newington, £43 2s. 6d.; Firs Home, Bournemouth, £19 3s. 4d.; St. Catherine's Home, Ventnor, £23 19s. 2d.; Friedenheim Hospital for the Dying, £297 1s. 8d.; Oxygen Home, Fitzroy Square, £35 9s. 2d.; Santa Claus Home, Highgate, £57 10s.; Free Home for Dying, Clapham, £124 11s. 8d.; St. Luke's House, Pembridge Square, W., £95 16s. 8d.

### 58 DISPENSARIES.

Battersea Provident Dispensary, £105 8s. 4d.; Billingsgate Medical Mission, £42 3s. 4d.; Blackfriars Provident Dispensary, £19 3s. 4d.; Bloomsbury Provident Dispensary, £15 6s. 8d.; Brixton, etc., Dispensary, £53 13s. 4d.; Brompton Provident Dispensary, £23 19s. 2d.; Buxton Street Dispensary, £28 15s.; Camberwell Provident Dispensary, £79 10s. 10d.; Camden Town Provident Dispensary, £15 6s. 8d.; Chelsea, Brompton and Belgrave Dispensary, £47 18s. 4d.; Chelsea Provident Dispensary, £13 8s. 4d.; Childs Hill Provident Dispensary, £15 6s. 8d.; City Dispensary, £47 18s. 4d.; City of London and East London Dispensary, £40 5s.; Clapham General and Provident Dispensary, £23; Deptford Medical Mission, £22 0s. 10d.; Eastern Dispensary, £28 15s.; East Dulwich Provident Dispensary, £43 2s. 6d.; Farringdon General Dispensary, £45 0s. 10d.; Finsbury Dispensary, £55 11s. 8d; Forest Hill Provident Dispensary, £42 3s. 4d.; Greenwich Provident Dispensary, £31 12s. 6d.; Hackney Provident Dispensary, £15 6s. 8d.; Hampstead Provident Dispensary, £52 14s. 2d.; Holloway and North Islington Dispensary, £51 15s.; Islington Dispensary, £59 8s. 4d.; Kensal Town Provident Dispensary, £14 7s. 6d.; Kilburn, Maida Vale and St. John's Wood Dispensary, £39 5s. 10d.; Kilburn Provident Medical Institution, £44 1s. 8d.; London Dispensary, Spitalfields, £12 9s. 2d.; London Medical Mission, Endell Street, £97 15s.; Margaret Street, for Consumption, £13 8s. 4d.; Metropolitan Dispensary, £53 13s. 4d.; Notting Hill Provident Dispensary, £14 7s. 6d.; Paddington Provident Dispensary, £31 12s. 6d.; Portland Town Dispensary, £13 8s. 4d.; Public Dispensary, Clare Market, W.C., £34 10s.; Queen Adelaide's Dispensary, £27 15s. 10d.; Royal General Dispensary, £22 0s. 10d; Royal Pimlico Provident Dispensary, £50 15s. 10d.; Royal South London Dispensary, £48 17s. 6d.; St. George's and St. James's Dispensary, £50 15s. 10d.; St. George's, Hanover Square, Provident Dispensary, £36 8s. 4d.; St. John's Wood Provident Dispensary, £45 0s. 10d.; St. Marylebone General Dispensary, £46; St. Pancras and Northern Dispensary,

£31 12s. 6d.: St. Pancras Medical Mission, £19 3s. 4d.; South Lambeth, Stockwell, and North Brixton Dispensary, £38 6s. 8d.; Stamford Hill, Stoke Newington Dispensary, £58 9s. 2d.; Tower Hamlets Dispensary, £44 1s. 8d.; Walworth Provident Dispensary, £14 7s. 6d.; Wandsworth Common Provident Dispensary, £11 10s.; Westbourne Provident Dispensary, £20 2s. 6d.; Western Dispensary, £61 6s. 8d.; Western General Dispensary, £115; Westminster General Dispensary, £40 5s.; Whitechapel Provident Dispensary, £35 9s. 2d.; Woolwich Provident Dispensary, £22 0s. 10d.

